# A Quantum Dot-Immunofluorescent Labeling Method to Investigate the Interactions between a Crinivirus and Its Whitefly Vector

**DOI:** 10.3389/fmicb.2013.00077

**Published:** 2013-04-04

**Authors:** James C. K. Ng

**Affiliations:** ^1^Department of Plant Pathology and Microbiology, Center for Disease Vector Research, University of CaliforniaRiverside, CA, USA

**Keywords:** fluorescence imaging, photostability, cibarium, foregut, quantum dot, *Lettuce infectious yellows virus*, *Bemisia tabaci*

## Abstract

Successful vector-mediated plant virus transmission entails an intricate but poorly understood interplay of interactions among virus, vector, and plant. The complexity of interactions requires continually improving/evaluating tools and methods for investigating the determinants that are central to mediating virus transmission. A recent study using an organic fluorophore (Alexa Fluor)-based immunofluorescent localization assay demonstrated that specific retention of *Lettuce infectious yellows virus* (LIYV) virions in the anterior foregut or cibarium of its whitefly vector is required for virus transmission. Continuous exposure of organic fluorophore to high excitation light intensity can result in diminished or loss of signals, potentially confounding the identification of important interactions associated with virus transmission. This limitation can be circumvented by incorporation of photostable fluorescent nanocrystals, such as quantum dots (QDs), into the assay. We have developed and evaluated a QD-immunofluorescent labeling method for the *in vitro* and *in situ* localization of LIYV virions based on the recognition specificity of streptavidin-conjugated QD_605_ (S-QD_605_) for biotin-conjugated anti-LIYV IgG (B-αIgG). IgG biotinylation was verified in a blot overlay assay by probing SDS-PAGE separated B-αIgG with S-QD_605_. Immunoblot analyses of LIYV using B-αIgG and S-QD_605_ resulted in a virus detection limit comparable to that of DAS-ELISA. In membrane feeding experiments, QD signals were observed in the anterior foregut or cibarium of virion-fed whitefly vectors but absent in those of virion-fed whitefly non-vectors. Specific virion retention in whitefly vectors corresponded with successful virus transmission. A fluorescence photobleaching assay of viruliferous whiteflies fed B-αIgG and S-QD_605_ vs. those fed anti-LIYV IgG and Alexa Fluor 488-conjugated IgG revealed that QD signal was stable and deteriorated approx. seven- to eight-fold slower than that of Alexa Fluor.

## Introduction

Affiliates of the genus *Crinivirus* (family *Closteroviridae*) infect diverse plant species (Wisler et al., 1998), and share common features, such as an exclusive tropism for phloem tissues and formation of filamentous virions that are transmitted in a semi-persistent manner by specific phloem-feeding whiteflies of the *Bemisia tabaci* species complex (Brown et al., 2000; Ng and Falk, 2006b; Dinsdale et al., 2010). The biology of semi-persistent transmission is described based on the classical observation that: (1) virions are acquired by the vector within minutes to hours; (2) acquired virions are retained in the vector from hours to days but are lost when the vector molts; (3) virions need not circulate through the vector or invade its salivary glands and other internal organs in order to be transmitted (Nault, 1997; Ng and Perry, 2004; Ng and Falk, 2006b). Current studies (as described below) have further advanced the concept that retention of virions in specific sites within the insect vector is critical in assuring virus transmission.

Our contributions to the understanding of the whitefly transmission of criniviruses have focused primarily on studies of *Lettuce infectious yellows virus* (LIYV), the type species of *Crinivirus*. These studies have benefited from the use of membrane feeding, a procedure that allows insects with piercing and sucking mouthparts to ingest virion-augmented artificial liquid diet sandwiched between a pair of stretched parafilm. Results from these studies provided concrete evidence that the whitefly *B. tabaci* biotype A can acquire and transmit LIYV virions purified from various sources, including cesium sulfate-sucrose density gradient-purified virions prepared from infected plants, and partially purified virions prepared from tobacco protoplasts inoculated with either virion RNAs or *in vitro* transcripts produced from cloned cDNAs corresponding to the viral genomic RNAs (Tian et al., 1999; Ng et al., 2004; Ng and Falk, 2006a). Results from these studies also suggested that transmission determinants of LIYV reside on the virion itself (Tian et al., 1999; Ng et al., 2004; Ng and Falk, 2006a), which contrasts with the aphid transmission of *Cauliflower mosaic virus* (CaMV) and viruses in the genus *Potyvirus*, where additional viral encoded proteins are needed to mediate virus transmission (Leh et al., 1999; Blanc et al., 2001; Pirone and Perry, 2002).

In a recent study in which immunofluorescent localization was used to analyze whiteflies that were sequentially fed LIYV virions, anti-LIYV virion IgG, and an organic fluorophore (Alexa Fluor 488)-conjugated goat anti-rabbit IgG, we found that upon uptake, LIYV virions were retained within the anterior foregut or cibarium of its specific vector *B. tabaci* biotype A, but not within that of the non-vector *B. tabaci* biotype B (Chen et al., 2011). Our study also demonstrated that specific virion retention in *B. tabaci* biotype A corresponded with the vector’s ability to successfully transmit LIYV (Chen et al., 2011). These observations are consistent with the notion that during acquisition feeding, the cibarium, a region in the alimentary tract posterior to the food canal, functions as a sucking pump to drive ingested plant sap (along with virions present in the sap) into the anterior foregut, the pharynx, and the esophagus of the insect; viruses that have established an intimate relationship with their whitefly vectors have the propensity to retain in these specific regions, whereupon they are eventually let go (egested) to be delivered into a plant during inoculation feeding (Harris, 1977).

During the course of our study, we observed that prolonged exposure of organic fluorophore to high intensity excitation light could result in diminished or loss of fluorescent signals in whitefly samples, particularly in situations where interactions were accompanied by weak signals. Indeed, photobleaching susceptibility is an inherent limitation associated with organic fluorophore-based analyses that can hamper observations requiring continuous exposure to blue light (Chan et al., 2002; Liu et al., 2005). In contrast, light-emitting semiconductor nanocrystals such as quantum dots (QDs) are less vulnerable to photobleaching because of their superior photostability (Alivisatos, 2004; Gao et al., 2005; Pinaud et al., 2006). Furthermore, they exhibit enhanced signal sensitivity due to a larger absorption cross section, larger stokes shift, and narrower fluorescence emission spectra when compared to organic fluorescent dyes and fluorescent proteins (Michalet et al., 2005; Pinaud et al., 2006). An additional advantage is that they are suitable for the detection of low copy numbers of biological molecules, or when these molecules are not densely concentrated in one location (Pinaud et al., 2006). As such, QDs are becoming a preferred label for the fluorescence imaging of biological samples. For example, they have been used extensively in mammalian cell biology studies and applications ranged from immunofluorescent localization of membrane receptors to the imaging of trafficking of cellular components by bio-conjugated QDs (Dahan et al., 2003; Wu et al., 2003; Chen and Gerion, 2004; Derfus et al., 2004; Lidke et al., 2004, 2007; Medintz et al., 2005; Bouzigues et al., 2007).

The work described in this paper pertains to the development of a QD-based strategy to examine LIYV-whitefly vector interactions occurring in a highly dynamic and turbulent region of the whitefly’s alimentary tract, where interacting components associated with virus transmission are constantly awash with an inflow and outflow of fluid. This study represents an inaugural demonstration of the effects of fluorescence photobleaching and the feasibility of the QD-labeling method as an improved system in the study of crinivirus-whitefly interaction. This system should also be applicable to the localization of other foregut-borne viruses that exhibit a similar mode of transmission as LIYV.

## Materials and Methods

### IgG preparation, biotin labeling and analysis

Polyclonal antibodies produced against LIYV virions were purified by ammonium sulfate precipitation and dialyzed using a 10 K molecular weight cut off (MWCO) membrane (Thermo-Pierce, Rockford, IL, USA), followed by DE52 cellulose (Whatman, England) anion exchange chromatography according to the methods of Harlow and Lane (1988). Fractions of IgG eluate were collected and quantified by UV spectrophotometry, assuming that an optical density of 1.35 corresponds to 1 mg/ml of IgG (Harlow and Lane, 1988). The IgG fractions with the highest OD readings were pooled and stored at 4°C until they were ready to be biotinylated with Sulfo-NHS-LC Biotin according to the manufacturer’s instructions (Thermo-Pierce, Rockford, IL, USA). Briefly, a 10 mg/ml of stock solution of Sulfo-NHS-LC Biotin was made immediately before use. Biotinylation was performed by incubating purified IgG with the stock solution of biotin in 1 × PBS (phosphate buffered saline; 3.2 mM Na_2_HPO_4_, 0.5 mM KH_2_PO_4_, 1.3 mM KCl, 135 mM NaCl, pH 7.4) on ice for 2 h, at a IgG:Sulfo-NHS-LC-Biotin molar ratio of 1:20. Excess biotin was removed by gel filtration chromatography using D-Salt™ Dextran columns according to the manufacturer’s instructions (Thermo-Pierce, Rockford, IL, USA). The collection and quantification of biotinylated IgG eluate were as described above.

Labeling of biotinylated-anti-LIYV virion IgG by streptavidin-conjugated QD_605_ (S-QD_605_) (Invitrogen) was performed in a blot overlay assay. Briefly, Biotinylated-LIYV IgG (B-αIgG) was separated by electrophoresis in a 12% SDS-PAGE at 100 V for 1.5 h, and transferred to a nitrocellulose membrane. The nitrocellulose membrane was incubated with 20 nM S-QD_605_ at room temperature for 1 h and then rinsed three times in wash buffer [1 × PBS with 0.3% (v/v) Tween 20]. Fluorescence imaging of the nitrocellulose membrane was performed using the Typhoon™ 9410 Variable Mode Imager (GE Healthcare, Sunnyvale, CA, USA) set at an excitation and emission wavelength of 457 and 610 nm, respectively, and a photomultiplier tube (PMT) voltage of 400 V.

### Double antibody sandwiched enzyme-linked immunosorbent assay and immunoblot analysis

Double antibody sandwiched-enzyme-linked immunosorbent assay (DAS-ELISA) (Clark and Adams, 1977) was used to determine the recognition and detection sensitivity of B-αIgG for purified LIYV virions. Hundred microliter of anti-LIYV serum diluted 1/500-fold in carbonate coating buffer (0.015 M Na_2_CO_3_ and 0.035 M NaHCO_3_, pH 9.6) was introduced into designated wells of a 96-well Polysorp™ microtiter plate (Nunc, USA). Hundred microliter of LIYV virions purified according to the methods of Tian et al. (1999), and diluted in sample buffer [1 × PBS with 0.05% (v/v) Tween 20, 2% (w/v) polyvinylpyrrolidone (PVP), pH 6] to concentrations ranging from 12 to 0.00012 ng/μl was added to each of the anti-LIYV serum coated wells. Following this, 100 μl of B-αIgG (approx. 0.6 mg/ml) diluted 1/500-fold in conjugate buffer [1 × PBS with 0.05% (v/v) Tween 20, 2% (w/v) PVP, 0.2% (w/v) bovine albumin serum (BSA), pH 7.4] was introduced into each of the designated wells. The final step involved the addition of NeutrAvidin-conjugated alkaline phosphatase (Thermo-Pierce, Rockford, IL, USA) at a 1/1000-fold dilution (2 μg/ml final concentration) in 1 × TBS (Tris buffered saline; 25 mM Trisbase, 1.3 mM KCl, 135 mM NaCl, pH 8) with 0.05% (w/v) Tween 20, 2% (w/v) PVP, and 0.2% (w/v) BSA to each of the designated wells. Plate incubation for each of the above steps was performed at 37°C for 2–3 h in a humid chamber. Plates were rinsed three times in wash buffer [1 × PBS with 0.05% (v/v) Tween 20] at the end of each step. After the final rinse, plates were added with 100 μl of 1-Step™ p-nitrophenyl phosphate substrate (Thermo Scientific, USA) at room temperature for 60 min for color development. The absorbance at 405 nm was measured in a Wallac Victor II Multilabel counter (Perkin Elmer, USA).

Immunoblot analysis of purified LIYV virions was as described previously (Tian et al., 1999), except that following the transfer of proteins to nitrocellulose membrane, the blot was incubated with B-αIgG at a 1/500-fold dilution in blocking buffer, followed by S-QD_605_ (10 nM), before it was analyzed by fluorescence imaging as described above.

### Whitefly transmission of LIYV virions and immunofluorescent localization assay

Three solutions were used for membrane feeding by whitefly vectors (*B. tabaci* biotype A) or non-vectors (*B. tabaci* biotype B) of LIYV. Solution 1: artificial diet [1 × TE (0.01 M Tris/HCl, 1 mM EDTA, pH 7.4) supplemented with 15% (w/v) sucrose and 1% (w/v) BSA] or artificial diet augmented with purified LIYV virions at a final concentration of 400 ng/μl (Klaassen et al., 1994; Tian et al., 1999; Ng et al., 2004). Solution 2: artificial diet augmented with B-αIgG at 1/500-fold dilution or anti-LIYV virion IgG at 1/362-fold dilution. Solution 3: artificial diet augmented with S-QD_605_ (20 nm final concentration) for detection of B-αIgG or with Alexa Fluor 488-conjugated goat anti-rabbit IgG (at 1/200-fold dilution; 10 μg/ml final concentration) for detection of anti-LIYV virion IgG. The experimental unit was a cage containing approx. 100 whiteflies (*B. tabaci* biotype A or B) taken randomly from the respective whitefly colony. In studies using biotype A (two independent experiments), there were altogether 11 and three replicates (cages) of virion-fed and diet-fed whiteflies, respectively (Table 2). In studies using biotype B (three independent experiments), there were altogether 18 and six cages of virion-fed and diet-fed whiteflies, respectively (Table 2). Following the ingestion of solution 1, 50 whiteflies from each cage were transferred to a lettuce plant for a 24 h inoculation feeding period (IAP). Plants were treated with an insecticide before being moved to an insect-proof greenhouse for symptom development. The remaining whiteflies in each cage (to be used for immunofluorescent localization) were fed solution 2, followed subsequently by solution 3. Whiteflies were all given a 10–12 h acquisition access period (AAP) for each of the three solutions. Whiteflies used for immunofluorescent localization were subjected to clearing after the first and the third solutions by allowing them to feed on artificial diet for 10–12 h to remove unbound components. These whiteflies were killed by freezing in −20°C and stored in this temperature until their heads were ready to be dissected for analysis. Whiteflies were dissected in deionized water (containing two drops of Tween 20 per 50 ml) on a microscope slide. Afterward, a cover slip was placed over the samples and sealed on all sides with ordinary nail polish, and the samples were observed by widefield fluorescence microscopy as described previously (Chen et al., 2011). A two-tailed Fisher’s exact test (JMP; SAS Institute) was used to evaluate the differences in percentage of: (1) virion-fed and diet-fed whiteflies (biotypes A and B) that contained fluorescence signals in their anterior foregut or cibarium, and (2) LIYV transmission by virion-fed biotype A and virion-fed biotype B.

### Fluorescence photobleaching assay

Heads dissected from whiteflies that were found by widefield fluorescence microscopy to contain fluorescent signals of Alexa Fluor 488 or QD 605 in the anterior foregut or cibarium were subjected to fluorescence photobleaching on a Leica SP5 confocal microscope, using a 20×/0.75NA water objective. The argon laser was set at 20% (for imaging) or 80% (for imaging and fluorescence photobleaching). The fluorescein isothiocyanate (FITC; 488 nm) argon laser line was set at 15% (for imaging) or 100% (for excitation and fluorescence photobleaching), and emission was collected between 500 and 530 nm (for Alexa Fluor signals), and between 603 and 608 nm (for QD signals). Images were acquired in xyt mode for 100 frames [1232 s (approx. 20 min)]. Fluorescence intensity values from three different regions of interest (ROIs), where QD or Alexa Fluor signals were detected, were collected over time (between *t* = 0 and 1232 s), and used to estimate the average fluorescence photobleaching rate using LAS AF software (Leica Microsystems).

## Results

### Biotinylation of anti-LIYV IgG and labeling with streptavidin-QD conjugate

To obtain biotinylated IgG produced against LIYV virions, anti-LIYV IgG was first purified from polyclonal anti-LIYV antiserum using ammonium sulfate precipitation and DE52 (Whatman, England) anion exchange chromatography (data not shown). Purified IgG from the most concentrated fraction (approx. 2.3 mg/ml) was biotinylated at an IgG:biotin molar ratio of 1:20, and purified by gel filtration chromatography. The biotinylated IgG eluate was collected and quantified, and fraction containing the highest IgG concentration (approx. 0.6 mg/ml) was used for all subsequent analyses and manipulations.

**Figure 1:**
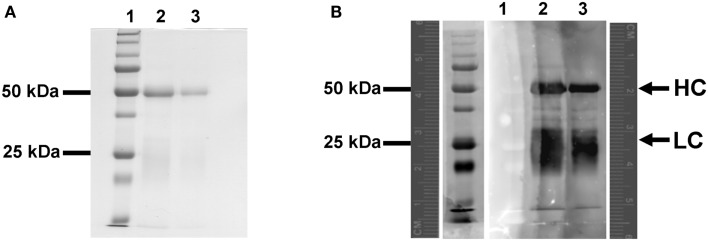
**Blot overlay assay of the interaction between biotinylated-anti-LIYV IgG and streptavidin quantum dot 605**. **(A)** SDS-polyacrylamide gel electrophoresis separation and Colloidal Coomassie blue staining of biotinylated-anti-LIYV IgG (B-αIgG). **(B)** Fluorescent imaging of a nitrocellulose membrane blotted with SDS-PAGE separated B-αIgG and overlaid with 10 nM streptavidin-conjugated QD605 (S-QD_605_). Lanes 1, low molecular weight prestained standards (the lane to the immediate left of lane 1 in **(B)** is an image of lane 1 captured under transmitted light); lanes 2 and 3, 3 and 1 μg of B-αIgG, respectively. A pair of fluorescence rulers is included in **(B)** to provide a reference for the migration distance of proteins detected under fluorescence and transmitted light. The positions of the 50 and 25 kDa prestained protein standards, and the heavy (HC) and light-chain (LC) polypeptides of B-αIgG are indicated.

A blot overlay assay was used to evaluate qualitatively the extent of biotinylation in the anti-LIYV IgG (B-αIgG) by probing with streptavidin-conjugated QD605 (S-QD_605_) (Invitrogen). B-αIgG was separated by electrophoresis in a 12% SDS-PAGE, transferred to nitrocellulose membrane, and overlaid with 20 nM S-QD_605_. Fluorescence analysis of the probed membrane revealed the presence of QD-labeled proteins of approx. 50 and 25 kDa, which corresponded with the molecular masses of the heavy- and light-chain polypeptides of IgG molecules, respectively (Harlow and Lane, 1988) (Figure 1).

### Evaluation of the recognition between biotinylated-anti-LIYV IgG and LIYV virions

IgG produced against LIYV virions routinely detects >1 ng of the LIYV major coat protein (CP) in immunoblot analysis (Ng et al., 2004). Thus, it was necessary and of interest to evaluate the virion detection sensitivity of B-αIgG to ensure that it was not affected by the biotinylation process. DAS-ELISA (see Materials and Methods for the details of the reacting components) was used to first ascertain the virion recognition specificity of B-αIgG (Table 1). The results revealed two findings: first, it further validated the success of IgG biotinylation, and provided clear evidence that B-αIgG was recognized by NeutrAvidin™ by way of biotin-NeutrAvidin™ non-covalent interaction, and that it also recognized and interacted with purified LIYV virions. Second, B-αIgG reacted positively with purified LIYV virions at concentrations ranging from 12 to 0.012 ng/μl (i.e., approx. 1200–1.2 ng per well in the microtiter plate) (Table 1), suggesting that its detection sensitivity was comparable to that of anti-LIYV IgG.

**Table 1:** **Virion recognition specificity of biotinylated-anti-LIYV IgG in DAS-ELISA**.

Virion concentration (ng/μl)	Absorbance values^a^ at 405 nm		Signal/noise
	Signal	Noise	
12	3.45	0.035	98.6
1.2	1.43	0.027	53.0
0.12	0.42	0.013	32.3
0.012	0.07	0.007	10.0
0.0012	0.02	0.008	2.5
0.00012	0.02	0.020	1.0

*^a^Average absorbance taken from wells incubated with LIYV virions (signal) and wells incubated with sample buffer (noise). Data are averages from three replicates. Reacting components in DAS-ELISA consisted of: coating antiserum (1:500 dilution), LIYV virions (at concentrations as indicated) or sample buffer, B-αIgG (1:500 dilution), and NeutrAvidin™ alkaline phosphatase (1:1000 dilution). Absorbance readings were taken 60 min after addition of the substrate, *p*-nitro phenyl phosphate*.

**Figure 2:**
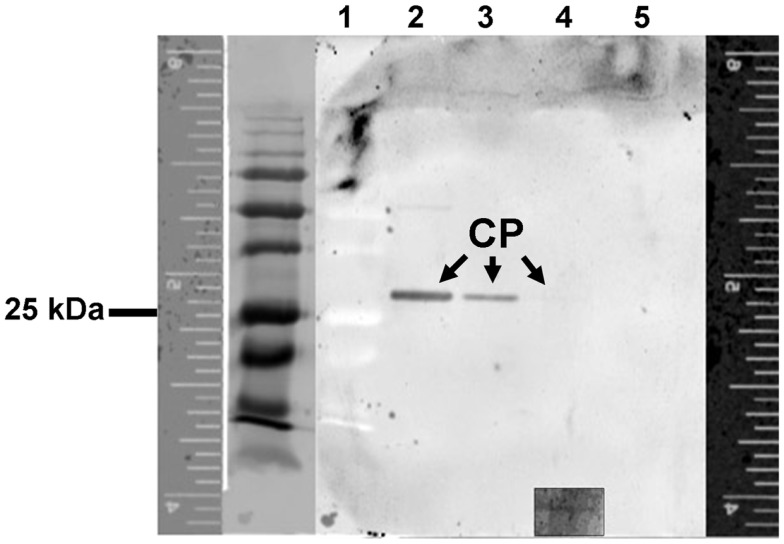
**Immunoblot and fluorescent imaging analysis to determine the recognition specificity between LIYV virions and biotinylated-anti-LIYV IgG *via* streptavidin quantum dot 605 binding**. Lane 1, low molecular weight prestained standards (the lane to the immediate left of lane 1 is an image of lane 1 captured under transmitted light); lanes 2–5, LIYV virions (1200, 120, 12, and 1.2 ng, respectively). Fluorescence rulers are included to provide a reference for the migration distance of proteins detected under fluorescence and transmitted light. The positions of the major coat protein (CP) of LIYV are indicated. The inset at the bottom of lane 4 is an image of the position in the membrane at which the CP was detected at a higher PMT of 500 V. The position of a 25 kDa prestained protein standard is as indicated.

Immunoblot analysis was used to further characterize the specific recognition of purified LIYV virions by B-αIgG under denaturing conditions. Following separation of virion proteins by SDS-PAGE and transfer to nitrocellulose membrane, the blot was probed with B-αIgG, followed by incubation with 10 nM S-QD_605_ (Figure 2), and LIYV CP was detected by the direct fluorescence imaging of the blot (Figure 2). The results indicated that B-αIgG reacted positively with purified LIYV virions at concentrations ranging from 120 to 0.12 ng/μl (i.e., approx. 1200–12 ng) (Figure 2).

### QD-immunofluorescent labeling and whitefly transmission of LIYV

**Figure 3:**
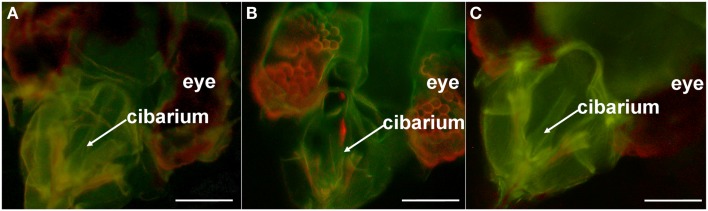
**Quantum dot (QD)-immunofluorescent localization of *Lettuce infectious yellows virus* in whitefly non-vector and whitefly vector by widefield fluorescence microscopy**. The dissected heads of: **(A)** non-vector (*B. tabaci* biotype B) and **(B)** vector (*B. tabaci* biotype A) whiteflies following the sequential acquisition feeding of artificial diet containing LIYV virions, biotinylated-anti-LIYV IgG (B-αIgG), and streptavidin-conjugated QD605 (S-QD_605_). **(C)** The dissected head of an unfed biotype A [i.e., it had not fed on artificial diet containing any of the components fed to the whiteflies in **(A,B)**]. Locations of the whitefly’s anatomical features (eye and cibarium) are included as points of reference. Bars represent 45 μm.

An organic fluorophore (Alexa Fluor 488)-based protocol was developed recently for the immunofluorescent localization of LIYV virions within whitefly vectors (Chen et al., 2011). Results from that study revealed that a green fluorescent signal was seen in the anterior foregut or cibarium of the whitefly vector, *B. tabaci* biotype A, following virion acquisition, and this localized signal corresponded consistently with successful virus transmission (Chen et al., 2011). As our repertoire of analyzes using organic fluorophore-based imaging grew, it became apparent that the use of high intensity excitation light for the prolonged examination of interactions that accompanied low fluorescent signals, such as those involving the acquisition of low concentrations of virions or LIYV capsid proteins, accelerated the susceptibility of fluorescent signal decay. Therefore, the development of a high excitation light intensity tolerant method of analysis that could circumvent the drawback of rapid fluorescent signal decay was highly desirable. As such, we designed and conducted new experiments to test a QD-based strategy of virion localization using B-αIgG and S-QD_605_. Here, we performed QD-immunofluorescent localization and whitefly transmission experiments to first determine if this approach gave reproducible results consistent with those observed using the Alexa Fluor 488-based protocol. Approx. 100 caged whitefly vectors (*B. tabaci* biotype A) or non-vectors (*B. tabaci* biotype B) were given two membrane feeding treatments–in the first treatment, whiteflies fed on a solution consisting of artificial diet augmented with 400 ng/μl of purified LIYV virions; in the second treatment, whiteflies fed on a solution consisting of artificial diet alone (i.e., no virions). Following acquisition feeding, half of the whiteflies in each treatment was transferred to non-infected

plants to determine virus transmissibility, while the remaining half of the whiteflies was fed artificial diet for several hours to flush out unbound virions (clearing). Afterward, the whiteflies were given sequential access to the following two solutions: artificial diet containing B-αIgG, and artificial diet containing S-QD_605_ (20 nM). After membrane feeding of the latter solution and clearing to flush out non-specifically bound antibodies, the heads of whiteflies were dissected and examined by widefield fluorescence microscopy (Table 2; Figure 3). In two independent experiments comparing vector whiteflies (Biotype A) fed on diet with or without virions, a red QD fluorescent signal was observed consistently in the anterior foregut or cibarium of 6–45% [at a combined total of 99 out of 376 (or approx. 26%)] of virion-fed vectors (Table 2; experiments 1 and 2; Figure 3), while signals were seen in 2–4% [at a combined total of 3 out of 131 (or approx. 2%)] of diet-fed vectors (Table 2; experiments 1 and 2). The difference in percentage of biotype A observed with QD fluorescence in the anterior foregut or cibarium in these two treatments was highly significant (*P* < 0.0001; Fisher’s exact test). The corresponding LIYV transmission success by the other half of the virion-fed vectors and diet-fed vectors that were allowed inoculation feeding on lettuce plants was 7 out of 11 plants (or approx. 64%) and 0 out of 3 plants, respectively (Table 2). In contrast, QD fluorescent signal was seen in the anterior foregut or cibarium of only 8 out of 688 (or approx. 1%) of virion-fed non-vectors (biotype B) [i.e., 99% did not show QD signals (Figure 3)], and 1 out of 255 (or approx. 0.4%) of diet-fed non-vectors (Table 2). The difference in percentage of biotype B observed with QD fluorescence in the anterior foregut or cibarium in these two treatments was highly insignificant (*P* = 0.4653; Fisher’s exact test). No corresponding LIYV transmission was observed in lettuce plants exposed to half of the virion-fed or diet-fed non-vector whiteflies (Table 2; experiments 3–5). The difference between the percentage of LIYV transmission by biotypes A and B was significant (*P* = 0.002; Fisher’s exact test).

**Table 2:** **Correspondence between quantum dot signals in the anterior foregut or cibarium of *Bemisia tabaci* and successful LIYV transmission**.

Experiment	*Bemisia tabaci* biotype A			Experiment	*Bemisia tabaci* biotype B		
	Virion-fed^a^	Diet-fed^b^	Transmission^c^		Virion-fed	Diet-fed	Transmission
1	4/28		−	3	1/25		−
	2/31		−		0/32		−
	10/36		+		0/28		−
	11/43		+		0/48		−
	10/42		+		1/42		−
		0/35	−		0/38		−
2	5/35		−			0/36	−
	13/31		−			1/34	−
	8/21		+	4	0/41		−
	13/33		+		0/30		−
	14/31		+		0/42		−
	9/45		+		1/40		−
		1/50	−		0/24		−
		2/46	−		0/32		−
						0/32	−
						0/43	−
				5	2/45		−
					2/45		−
					1/46		−
					0/43		−
					0/46		−
					0/41		−
						0/40	−
						0/40	−

*^a^LIYV virions were diluted to a concentration of approx. 400 ng/μl in artificial diet and presented to 100 whiteflies (*B. tabaci* biotypes A or B) for acquisition feeding, following which approx. 50 whiteflies were transferred to a target plant for inoculation feeding. The remaining (approx. 50) whiteflies were fed diet augmented with biotinylated-LIYV IgG and streptavidin-conjugated QD605. The heads of these whiteflies were excised and analyzed by widefield fluorescence microscopy. Fluorescence labeling was scored as the number of heads detected with QD signal in the foregut or cibarium over the total number of heads examined*.

*^b^The same treatment as above, except that whiteflies were fed artificial diet containing no virions*.

*^c^+ and − indicate infection and no infection, respectively, of target plants following inoculation feeding by whiteflies*.

In all cases, no signal was observed in any other regions of the food canal (Figure 3) and mouthparts, including the distal end of the maxillary stylets (not shown). The red fluorescence seen in the eyes of both the virion-fed biotypes B (non-vector) and A (vector) was autofluorescence (Figures 3A,B), as similar fluorescence was also observed in the eyes of biotypes B (not shown) and A that did not feed on diet augmented with virions, B-αIgG, and S-QD_605_. (Figure 3C).

These data demonstrated that the QD-based immunofluorescent localization approach is applicable to the study of LIYV-whitefly vector interaction. Consistent with our previous study (Chen et al., 2011), these results suggested that LIYV virions are retained in the anterior foregut or cibarium of the whitefly vector, *B. tabaci* biotype A, and that specific retention in these locations corresponded with *B. tabaci* biotype A-mediated transmission of the virus.

### Stability of QD-immunofluorescent labeling determined by fluorescence photobleaching

**Figure 4:**
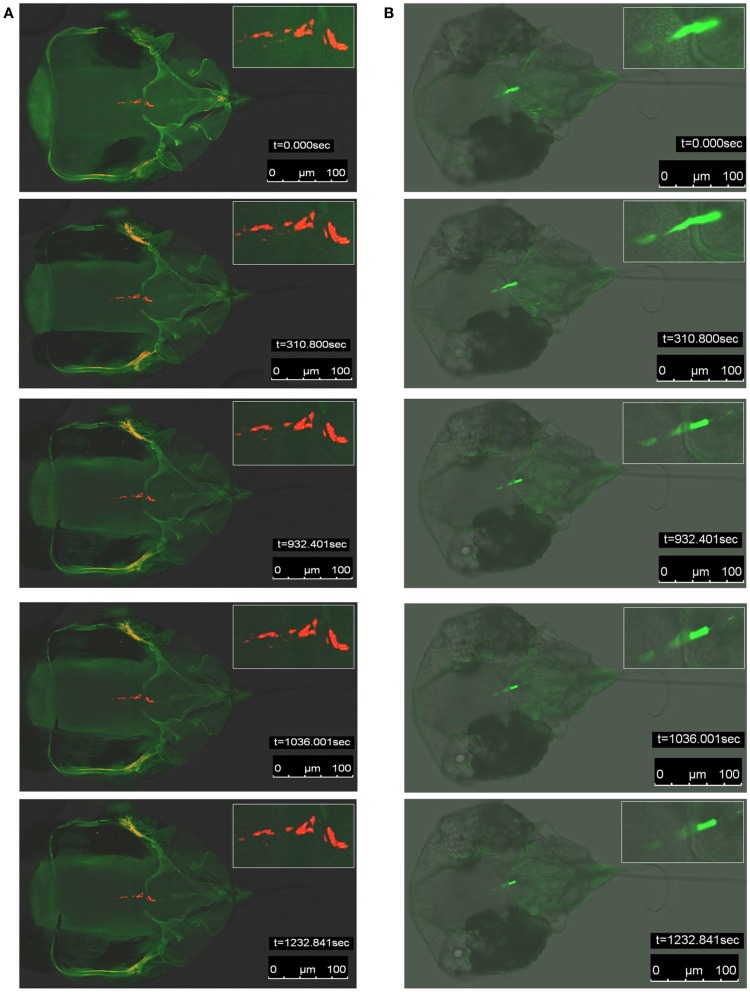
**Fluorescence photobleaching of specific quantum dot (QD) or Alexa Fluor signals in viruliferous whitefly (*B. tabaci* biotype A) vectors**. **(A)** Snapshots of five representative confocal laser scanning microscopy (CLSM) images of fluorescence photobleaching in QD-immunofluorescent labeled (red signals) whitefly vectors fed artificial diet augmented with LIYV virions, biotinylated-anti-LIYV IgG (B-αIgG), and streptavidin-conjugated QD605 (S-QD_605_). **(B)** Snapshots of five CLSM images of fluorescence photobleaching in Alexa Fluor 488-immunofluorescent labeled (green signals) whitefly vectors fed artificial diet augmented with LIYV virions, anti-LIYV virion IgG, and Alexa Fluor 488-conjugated goat anti-rabbit IgG. The Inset in each snapshot is an enlarged image of the region in which QD or Alexa Fluor signals were detected. The time intervals (*t* = 0.000, 310.800, 932.401, 1036.001, 1232.841 s) at which snapshots were taken and bars representing 100 μm are indicated in each snapshot.

We next determined if the QD signal observed in whitefly vectors could hold up to the rigors of continuous high intensity excitation light exposure better than Alexa Fluor signal. This objective was achieved by conducting fluorescence photobleaching experiments in parallel for viruliferous whiteflies fed B-αIgG, and S-QD_605_ vs. those fed anti-LIYV IgG and Alexa Fluor 488-conjugated goat anti-rabbit IgG. In these experiments, QD or Alexa Fluor fluorescent signal decay observed in the anterior foregut or cibarium of viruliferous whiteflies was measured for 1232 s (approx. 20 min), which facilitated the estimation of the photobleaching rate. We used the ROIs tool in the LAS AF software (Leica Microsystems) to define a 15 μm^2^ circle, and used this defined area to mark three ROIs (not shown) in the anterior foregut or cibarium, where QD or Alexa Fluor fluorescent signals were observed. The signal intensity values (not shown) quantified in these ROIs were then used to estimate the rate of fluorescent signal decay over the 1232-s assay period. When the results were analyzed and compared, we noted a considerable difference in photobleaching rate between QD and Alexa Fluor fluorescence. In the representative analyses shown in Figure 4A and Movie S1A in Supplementary Material, QD fluorescent decay was not noticeable by visual inspection throughout the entire duration of the assay. In contrast, the decay of Alexa Fluor signal became visually discernible from *t* = 350 s to the end of the assay at *t* = 1232 s (Figure 4B; Movie S1B in Supplementary Material). An estimate of the average rate of photobleaching sampled from the marked ROIs was approx. 2 × 10^−2^/s and 1.6 × 10^−1^/s for QD and Alexa Fluor signals, respectively, i.e., about an eightfold difference. Taken together, these qualitative and quantitative data provided evidence that fluorescence emitted by QD within the anterior foregut or cibarium of viruliferous whitefly vectors was stable and deteriorated at a much slower rate compared to that of Alexa Fluor.

## Discussion

fillskip0ptFor over 20 years, investigations on vector retention sites of foregut-borne, semi-persistently transmitted plant viruses have relied primarily on studies involving the transmission electron microscopy (TEM) analyses of serial thin sections of viruliferous insect vectors. For example, TEM analyses had shown that virions of several semi-persistently transmitted viruses (*Anthriscus yellows virus*, *Parsnip yellow fleck virus*, and *Maize chlorotic dwarf virus*), all unrelated to criniviruses, were bound to sites within the foregut of their respective insect vectors following virus acquisition (Murant et al., 1976; Childress and Harris, 1989; Ammar and Nault, 1991). One major disadvantage of the TEM approach is that it is tedious (involving long periods of labor), and requires a high level of skills and experience to perform, which could be the reasons underlying the hitherto limited number of reports in the literature. To overcome the challenges that are limiting our understanding of the processes occurring in the insect vector following virus uptake, we developed an Alexa Fluor-based immunofluorescent localization assay, the predecessor of the QD-immunofluorescent localization assay developed in the current study, and have used it to characterize the interactions between LIYV and its whitefly vector (Chen et al., 2011). Results obtained from the Alexa Fluor-protocol and the QD-protocol (discussed in detail below) have shown that both are innovative approaches and, as a whole, enabled us to test and prove the hypotheses that have already been alluded to in the preceding sections: i.e., that upon uptake, virions of LIYV are retained in specific sites in the anterior foregut or cibarium of the whitefly vector, and virions retained in these specific sites are positioned strategically to take advantage of the egestion process to facilitate their transmission into a plant during inoculation feeding. When higher concentrations (>100 ng/μl) of virions are used in membrane feeding experiments involving the Alexa Fluor-based protocol, continuous areas of strong fluorescent signals are typically found to occupy the anterior foregut or cibarium of whitefly vectors similar to the results shown in Figure 4B. However, in virion acquisition and transmission experiments that involve low virion concentrations (≤10 ng/μl), the signals may not be easily discernible. For example, we are currently conducting studies to determine the range of virion concentrations that would support virion retention and transmission by whitefly vectors. At the lower end of the concentration range, weak signals prevail (Chen and Ng, unpublished data), which may require fluorophores to be exposed to excitation light for a longer duration and/or at a higher intensity in order for signals to be visualized unmistakably. Under such circumstances, fluorescent signals emitted from organic fluorophores would be susceptible to accelerated photobleaching, as has been demonstrated by our fluorescence photobleaching assay (Figure 4B; Movie S1B in Supplementary Material), thereby biasing the results in favor of no virion retention.

Our goal of the current study was to overcome the limitation of rapid fluorescence decay through the development of a QD-immunofluorescent localization system suitable for use with assays requiring continuous exposure of samples to high excitation light intensity. We began the study with preparative steps leading to the biotinylation of an anti-LIYV IgG. Successful biotinylation of this IgG, B-αIgG, was confirmed by testing its recognition of and affinity for virions, and streptavidin-conjugated QD605 (S-QD_605_) or NeutrAvidin™-alkaline phosphatase using a combination of blot overlay assay (Figure 1), immunoblot detection (Figure 2), and functional detection by DAS-ELISA (Table 1). The virion detection limit of B-αIgG was between 12 and 1.2 ng, depending on the assay employed, and was comparable to that of non-biotinylated-LIYV IgG (Ng et al., 2004).

We then exploited the high affinity of biotin for streptavidin (Green, 1963) by using them as tools for the *in situ* visualization of virion retention within whitefly vectors (Figures 3 and 4). As discussed above, evidence has been presented here to demonstrate that the presence of QD signals in the anterior foregut or cibarium of viruliferous whitefly vectors corresponds to successful LIYV transmission (Table 2). The QD signals seen in these virion retention sites could not be due to the non-specific binding of B-αIgG and S-QD_605_, especially since most vectors and non-vectors that fed on artificial diet alone (i.e., no virions) followed sequentially by artificial diet containing B-αIgG and S-QD_605_ did not contain signals in these locations (Table 2). Thus, data obtained using QD-immunofluorescent localization were consistent with those obtained using the Alexa Fluor-based protocol (Chen et al., 2011). As with the latter protocol, the QD-immunofluorescent localization assay also contains an inherent limitation. In order for a positive signal to be seen at the retention sites, the whitefly has to acquire all interacting components (i.e., virions, B-αIgG and S-QD_605_) during membrane feeding. Thus, no signal will be observed in a whitefly that has acquired only one or two of the three components. Nonetheless, this approach has proven reliable in that a substantial number of virion-fed whiteflies clearly showed signals in their foreguts compared to fewer false positives seen in diet-fed vectors, as well as virion-fed or diet-fed non-vectors (Table 2). Given the inherent variability in the acquisition of the individual components, our estimate of 26% of biotype A that showed specific QD signals did not appear to deviate considerably from the 39% previously observed using the Alexa Fluor procedure (Table 2) (Chen et al., 2011). In the absence of virions (i.e., when whiteflies were fed only B-αIgG and S-QD_605_ in this study, or only anti-LIYV IgG and Alexa Fluor 488-conjugated goat anti-rabbit IgG in the previous study), the level of false positives, were also comparable, ranging from 2 to 4% and 0 to 2%, respectively (Table 2) (Chen et al., 2011). In the case of biotype B (the non-vector), the level of false positives remained consistent for both the QD and Alexa Fluor procedures whether whiteflies were fed all three components (false positive was 1% for both procedures), or only the second and third components (0.4 vs. 0%, respectively) (Table 2) (Chen et al., 2011). Recent studies aimed at understanding *Banana bunchy top virus* (BBTV) tropism within aphid vectors have used a biotin-streptavidin-Alexa Fluor-based immunofluorescent assay for the *in situ* localization of virus within the gut and salivary gland tissues of viruliferous aphids (Bressan and Watanabe, 2011; Watanabe and Bressan, 2013). The principle underlying this detection method and ours is similar in that both involve signal amplification. However, because the *in situ* BBTV localization approach is applied after insect tissues of viruliferous aphids have been dissected, and involves an extra reaction step (primary virus specific antibody, biotin-conjugated goat anti-rabbit antibody, followed by Alexa Fluor 488-conjugated streptavidin), it is unclear as to the utility of this approach in facilitating the *in situ* localization of LIYV and other foregut-borne viruses. Studies are currently being attempted to efficiently couple QD or other photostable nanocrystals and equivalents to virion specific IgG that could then be incorporated into the immunofluorescent localization assay to cut down the number of steps in the assay to two, thereby streamlining and improving the versatility of the process. In addition, it would pave the way for the development of a system capable of labeling the virions of more than one virus concurrently; thus allowing us to address questions concerning the co-retention of multiple viruses.

One novelty of this work was the use of photobleaching to compare the stability of QD605 and Alexa Fluor 488 within the virion retention sites in the anterior foregut or cibarium of viruliferous whitefly vectors. Results from the study showed that the QD-immunofluorescent localization protocol provides sensitive labeling of LIYV virions in these retention sites, while exhibiting high fluorescence photostability over that of Alexa Fluor. A fundamental issue concerning the use of QD in immunostaining of tissues is the inherent size of the nanocrystals and hence potential difficulty of penetration and wash out. This does not apply in our case because the virion-antibody interaction sites are in an open lumen (the alimentary tract). Therefore, QD is as effective here as it is when used in surface labeling of cells for flow cytometry. Thus, due to the sensitivity of QD and its resistance to photobleaching, QD-based approaches should be particularly well-suited for localization studies of foregut-borne viruses involving the use of procedures that require prolonged exposure to high intensity excitation light, or when only low amounts of virions/viral encoded proteins are present.

## Conflict of Interest Statement

The authors declare that the research was conducted in the absence of any commercial or financial relationships that could be construed as a potential conflict of interest.

## Supplementary Material

The Supplementary Material for this article can be found online at: http://www.frontiersin.org/Virology/10.3389/fmicb.2013.00077/abstract

## References

[B1] Alivisatos P. (2004). The use of nanocrystals in biological detection. Nat. Biotechnol. 22, 47–52 10.1038/nbt927 14704706

[B2] Ammar E. D. Nault L. R. (1991). *Maize chlorotic dwarf virus-*like particles associated with the foregut in vector and non-vector leafhopper species. Phytopathology 81, 444–448 10.1094/Phyto-81-444

[B3] Blanc S. Hébrard E. Drucker M. Froissart R. (2001). “Molecular basis of vector transmission: caulimoviruses,” in Virus-Insect-Plant Interactions, eds Harris K. Smith O. P. Duffus J. E. (San Diego: Academic Press), 143–166

[B4] Bouzigues C. Morel M. Triller A. Dahan M. (2007). Asymmetric redistribution of GABA receptors during GABA gradient sensing by nerve growth cones analyzed by single quantum dot imaging. Proc. Natl. Acad. Sci. U.S.A. 104, 11251–11256 10.1073/pnas.0702536104 17592112 PMC2040885

[B5] Bressan A. Watanabe S. (2011). Immunofluorescence localisation of *Banana bunchy top virus* (family Nanoviridae) within the aphid vector, *Pentalonia nigronervosa*, suggests a virus tropism distinct from aphid-transmitted luteoviruses. Virus Res. 155, 520–525 10.1016/j.virusres.2010.12.005 21167229

[B6] Brown J. K. Perring T. M. Cooper A. D. Bedford I. D. Markham P. G. (2000). Genetic analysis of Bemisia (Hemiptera: Aleyrodidae) populations by isoelectric focusing electrophoresis. Biochem. Genet. 38, 13–25 10.1023/A:1001806702292 10862356

[B7] Chan W. C. Maxwell D. J. Gao X. Bailey R. E. Han M. Nie S. (2002). Luminescent quantum dots for multiplexed biological detection and imaging. Curr. Opin. Biotechnol. 13, 40–46 10.1016/S0958-1669(02)00282-3 11849956

[B8] Chen A. Y. S. Walker G. P. Carter D. Ng J. C. K. (2011). A virus capsid component mediates virion retention and transmission by its insect vector. Proc. Natl. Acad. Sci. U.S.A. 108, 16777–16782 10.1073/pnas.1017677108 21930903 PMC3189079

[B9] Chen F. Gerion D. (2004). Fluorescent CdSe/ZnS nanocrystal-peptide conjugates for long-term, nontoxic imaging and nuclear targeting in living cells. Nano Lett. 4, 1827–1832 10.1021/nl049170q

[B10] Childress S. A. Harris K. F. (1989). Localization of virus-like particles in the foreguts of viruliferous *Graminella nigrifrons* leafhoppers carrying the semi-persistent *Maize chlorotic dwarf virus*. J. Gen. Virol. 70, 247–251 10.1099/0022-1317-70-1-247

[B11] Clark M. F. Adams A. N. (1977). Characteristics of the microplate method of enzyme-linked immunosorbent assay for the detection of plant viruses. J. Gen. Virol. 34, 475–483 10.1099/0022-1317-34-3-475 323416

[B12] Dahan M. Levi S. Luccardini C. Rostaing P. Riveau B. Triller A. (2003). Diffusion dynamics of glycine receptors revealed by single-quantum dot tracking. Science 302, 442–445 10.1126/science.1088525 14564008

[B13] Derfus A. M. Chan W. C. W. Bhatia S. N. (2004). Intracellular delivery of quantum dots for live cell labeling and organelle tracking. Adv. Mater. 16, 961–966 10.1002/adma.200306111

[B14] Dinsdale A. Cook L. Riginos C. Buckley Y. M. De Barro P. (2010). Refined global analysis of *Bemisia tabaci* (Hemiptera: Sternorrhyncha: Aleyrodoidea: Aleyrodidae) mitochondrial oxidase 1 to identify species level genetic boundaries. Ann. Entomol. Soc. Am. 103, 196–208 10.1603/AN09061

[B15] Gao X. Yang L. Petros J. A. Marshall F. F. Simons J. W. Nie S. (2005). In vivo molecular and cellular imaging with quantum dots. Curr. Opin. Biotechnol. 16, 63–72 10.1016/j.copbio.2004.11.003 15722017

[B16] Green N. M. (1963). Avidin. 1. The use of (14-C) biotin for kinetic studies and for assay. Biochem. J. 89, 585–591 14101979 10.1042/bj0890585 PMC1202466

[B17] Harlow E. Lane D. (1988). Antibodies: A Laboratory Manual. New York: Cold Spring Harbor

[B18] Harris K. F. (1977). “An ingestion-egestion hypothesis of transmission,” in Aphids as Virus Vectors, eds Harris K. F. Maramorosch K. (New York: Academic Press), 165–219

[B19] Klaassen V. A. Boeshore M. Dolja V. V. Falk B. W. (1994). Partial characterization of the *lettuce infectious yellows virus* genomic RNAs, identification of the coat protein gene and comparison of its amino acid sequence with those of other filamentous RNA plant viruses. J. Gen. Virol. 75(Pt 7), 1525–1533 10.1099/0022-1317-75-7-1525 8021583

[B20] Leh V. Jacquot E. Geldreich A. Hermann T. Leclerc D. Cerutti M. (1999). Aphid transmission of cauliflower mosaic virus requires the viral PIII protein. EMBO J. 18, 7077–7085 10.1093/emboj/18.24.7077 10601029 PMC1171770

[B21] Lidke D. S. Nagy P. Heintzmann R. Arndt-Jovin D. J. Post J. N. Grecco H. E. (2004). Quantum dot ligands provide new insights into erbB/HER receptor-mediated signal transduction. Nat. Biotechnol. 22, 198–203 10.1038/nbt929 14704683

[B22] Lidke D. S. Nagy P. Jovin T. M. Arndt-Jovin D. J. (2007). Biotin-ligand complexes with streptavidin quantum dots for in vivo cell labeling of membrane receptors. Methods Mol. Biol. 374, 69–79 17237530 10.1385/1-59745-369-2:69

[B23] Liu T. Liu B. Zhang H. Wang Y. (2005). The fluorescence bioassay platforms on quantum dots nanoparticles. J. Fluoresc. 15, 729–733 10.1007/s10895-005-2980-5 16341790

[B24] Medintz I. L. Uyeda H. T. Goldman E. R. Mattoussi H. (2005). Quantum dot bioconjugates for imaging, labelling and sensing. Nat. Mater. 4, 435–446 10.1038/nmat1390 15928695

[B25] Michalet X. Pinaud F. F. Bentolila L. A. Tsay J. M. Doose S. Li J. J. (2005). Quantum dots for live cells, in vivo imaging, and diagnostics. Science 307, 538–544 10.1126/science.1104274 15681376 PMC1201471

[B26] Murant A. F. Roberts I. M. Elnagar S. (1976). Association of virus-like particles with foregut of aphid *Cavariella-aegopodii* transmitting semi-persistent viruses *Anthriscus yellows* and *Parsnip yellow fleck*. J. Gen. Virol. 31, 47–57 10.1099/0022-1317-31-1-47

[B27] Nault L. R. (1997). Arthropod transmission of plant viruses: a new synthesis. Ann. Enotomol. Soc. Am. 90, 522–541

[B28] Ng J. C. Falk B. W. (2006a). *Bemisia tabaci* transmission of specific *Lettuce infectious yellows virus* genotypes derived from in vitro synthesized transcript-inoculated protoplasts. Virology 352, 209–215 10.1016/j.virol.2006.04.020 16750548

[B29] Ng J. C. Falk B. W. (2006b). Virus-vector interactions mediating nonpersistent and semipersistent transmission of plant viruses. Annu. Rev. Phytopathol. 44, 183–212 10.1146/annurev.phyto.44.070505.143325 16602948

[B30] Ng J. C. Tian T. Falk B. W. (2004). Quantitative parameters determining whitefly (*Bemisia tabaci*) transmission of *Lettuce infectious yellows virus* and an engineered defective RNA. J. Gen. Virol. 85, 2697–2707 10.1099/vir.0.80189-0 15302963

[B31] Ng J. C. K. Perry K. L. (2004). Transmission of plant viruses by aphid vectors. Mol. Plant Pathol. 5, 505–511 10.1111/j.1364-3703.2004.00240.x 20565624

[B32] Pinaud F. Michalet X. Bentolila L. A. Tsay J. M. Doose S. Li J. J. (2006). Advances in fluorescence imaging with quantum dot bio-probes. Biomaterials 27, 1679–1687 10.1016/j.biomaterials.2005.11.018 16318871 PMC3073483

[B33] Pirone T. P. Perry K. L. (2002). “Aphids-nonpersistent transmission,” in Adv Bot Res, Vol. 36, ed. Preston R. D. (London: Academic Press), 1–19

[B34] Tian T. Rubio L. Yeh H. H. Crawford B. Falk B. W. (1999). *Lettuce infectious yellows virus*: in vitro acquisition analysis using partially purified virions and the whitefly *Bemisia tabaci*. J. Gen. Virol. 80(Pt 5), 1111–1117 10355756 10.1099/0022-1317-80-5-1111

[B35] Watanabe S. Bressan A. (2013). Tropism, compartmentalization and retention of *Banana bunchy top virus* (Nanoviridae) in the aphid vector *Pentalonia nigronervosa*. J. Gen. Virol. 94, 209–219 10.1099/vir.0.047308-0 23015741

[B36] Wisler G. C. Duffus J. E. Liu H. Y. Li R. H. (1998). Ecology and epidemiology of whitefly-transmitted closteroviruses. Plant Dis. 82, 270–280 10.1094/PDIS.1998.82.3.270 30856856

[B37] Wu X. Liu H. Liu J. Haley K. N. Treadway J. A. Larson J. P. (2003). Immunofluorescent labeling of cancer marker Her2 and other cellular targets with semiconductor quantum dots. Nat. Biotechnol. 21, 41–46 10.1038/nbt819 12459735

